# Relationship between the invasion of lymphocytes and cytokines in the tumor microenvironment and the interval after single brachytherapy hypofractionated radiotherapy and conventional fractionation radiotherapy in non-small cell lung Cancer

**DOI:** 10.1186/s12885-020-07403-1

**Published:** 2020-09-17

**Authors:** Lin Li, Hong Cheng Yue, Yun Wei Han, Wei Liu, Liang Geng Xiong, Jian Wen Zhang

**Affiliations:** 1 Department of Oncology, The Affiliated Hospital of Southwest Medical University, Luzhou, Sichuan 646000 People’s Republic of China; 2 Department of Oncology, Central Hospital of Bazhong, Bazhong, Sichuan 636000 People’s Republic of China; 3 Nuclear Medicine and Molecular Imaging Key Laboratory of Sichuan Province, Luzhou, Sichuan People’s Republic of China; 4 Department of Oncology, People’s Hospital of Dazu District, Banan District Chongqing, 402360 People’s Republic of China; 5 Department of Oncology, People’s Hospital of Chongqing Banan District, Banan District Chongqing, 401320 People’s Republic of China; 6 Academician (Expert) workstation of Sichuan Province, Luzhou, Sichuan People’s Republic of China

**Keywords:** Brachytherapy, Conventional fractionation radiotherapy, Cytokine, Hypofractionated radiotherapy, Lymphocyte, Non-small cell lung cancer

## Abstract

**Background:**

The effect of brachytherapy on lymphocytes and cytokines in the tumor microenvironment is unclear. This study aimed to analyze the relationship between the invasion of lymphocytes and cytokines in the tumor microenvironment and the interval after single brachytherapy hypofractionated radiotherapy (SBHFRT) and conventional fractionation radiotherapy (CFRT) in non-small cell lung cancer (NSCLC).

**Methods:**

Lewis tumor-bearing mice were randomly divided into control, CFRT, and SBHFRT groups. On days 7 and 14 after radiation, the expression levels of CD86^+^, CD4^+^, CD8^+^, and Foxp3^+^ cells, and levels of Ki-67^+^ protein were detected by immunohistochemistry, and the tumor necrosis rate was calculated. Following this, the levels of interleukin-10 (IL-10), IL-12, and interferon-γ (IFN-γ) were detected by enzyme-linked immunosorbent assay. The apoptosis rate was evaluated via flow cytometry. The tumor volume and tumor growth inhibition rate (TGIR) were calculated on day 14. Tumor metabolism was assessed via 18F-FDG micropositron emission tomography/computer tomography.

**Results:**

The tumor volume reduced by 22.0% and TGIR increased by 92.2% (*p* < 0.05) in the SBHFRT group. Further, on days 7 and 14 after radiation, tumor metabolism, Ki-67^+^ and Foxp3^+^ expression levels, and IL-10 levels were lower, and tumor necrosis and apoptosis rates; CD86^+^, CD4^+^, and CD8^+^ expression levels; and IL-12 and IFN-γ levels were higher in the SBHFRT group than in the CFRT group, particularly on day 7.

**Conclusion:**

SBHFRT could lead to more accumulation of dendritic cells, anti-tumor lymphocytes, and cytokines, and further reduce the aggregation of immunosuppressive lymphocytes and cytokines in the tumor microenvironment compared with CFRT, and the difference was the most obvious on day 7 after radiation. The clinical significance of the findings remains to be further verified.

## Background

Lung cancer, particularly non-small cell lung cancer (NSCLC), is a common malignancy, in which radiotherapy plays an important therapeutic role [1]. The radiotherapy model for malignancy has transformed from conventional fraction radiotherapy (CFRT) to hypofractionated radiotherapy, which is now considered to be a new and highly effective mode of radiation therapy [1, 2]. Stereotactic body radiotherapy (SBRT) and stereotactic radiosurgery are common procedures in hypofractionated radiotherapy, especially SBRT, which is widely performed for lung cancer, colorectal cancer, liver cancer, and prostate cancer, and shows considerable improvement in the radiotherapeutic outcomes [3–8].

Immunotherapy has recently emerged as a highly effective and novel therapeutic modality, and is now gaining popularity worldwide for cancer therapy [9]. Immunocheckpoint inhibitors, such as pembrolizumab and ipilimumab, are widely used for melanoma, NSCLC, and pancreatic cancer [10–13]. The core functionality of immunotherapy relies on the fact that there are enough anti-tumor cells in the tumor tissue, and the destruction of immune cells in tumor tissues will lead to the failure of immunotherapy [14, 15]. Accordingly, improving and enhancing immune cells in tumor tissues, especially CD8^+^ T lymphocytes, is the key to successful tumor immunotherapy [15].

The relationship between radiation and immunity is complicated. Studies have now shown that in addition to inducing lethal DNA damage in tumor and stromal cells, radiation can alter interactions of tumor cells with their microenvironment, and these effects on the tumor microenvironment vary with dose and fractionation schedules [16, 17]. Hypofractionated radiotherapy can stimulate the immune system. NSCLC patients undergoing SBRT have been reported to show increased levels of CD8^+^ T cells and interferon-γ (IFN-γ), and decreased levels of inhibitory T reg cells [18]. In colon cancer, SBRT activates dendritic cells (DCs) and induces immune cell infiltration in tumors and migration of immune cells to tumors [17].

The use of single brachytherapy hypofractionated radiotherapy (SBHFRT) in advanced NSCLC has rarely been reported in the literature, and there is no study on the relationship of SBHFRT with immune cells. The results of our previous clinical studies have shown that the effective rate and 2-year overall survival after SBHFRT in patients with NSCLC were 92.3 and 67%, respectively, with a median survival period of 22.5 months [19]. Whether this outcome, in addition to the physical role of radioactive rays, is associated with immune cells and cytokines, particularly anti-tumor lymphocytes and cytokines in the tumor microenvironment, it is unknown. The main purpose of this study was to investigate the relationship between the invasion of lymphocytes and cytokines in the tumor microenvironment and the interval after SBHFRT and CFRT for NSCLC through animal experiments, and to provide the basis for radiotherapy combined with immunotherapy in NSCLC.

## Methods

### Establishment of the tumor-bearing mouse model

Resuscitation and culture of Lewis cell line of NSCLC (department of oncology, affiliated hospital of southwest medical university) was performed at the medical laboratory center (affiliated hospital of southwest medical university). The Lewis cell line stored at − 80 °C in a refrigerator was thawed in a water bath at 37 °C, and centrifuged at 1000 RPM for 1 min. The supernatant was discarded, 1.0 mL of the medium was added to the frozen storage tube, and the cell sediment at the bottom of the tube was mixed in to a uniform cell suspension. Subsequently, the cell suspension was transferred into a 25cm^2^ culture bottle and 3 mL of the medium was added, followed by incubation at a constant temperature of 37 °C with 5% CO_2_. The cell growth was observed under a phase contrast microscope every day, and the medium was changed every 2 days.

Healthy C57BL/16 female mice (total 36; aged 4–5 weeks; weighing 16-22 g; Chongqing tianxinhuafu biotechnology company, China. Certificate No: 11401300024918; with SPF feeding at the medical laboratory animal center, the affiliated hospital of southwest medical university) were acclimatized for at least a week under standard conditions of 24 ± 2 °C and 50 ± 10% relative humidity before study enrollment.

Lewis cell line (1 × 10^7^/mL) cells in their exponential growth phase were inoculated via a subcutaneous injection into the right hind limb of healthy C57BL/16 female mice after disinfecting their skin with 75% medical alcohol, and mental, dietary, weight, behavioral, and tumor volume changes in these mice were recorded daily. Experiments were performed when the diameter of the transplanted tumor was approximately 8–10 mm. All animal experiments were approved by the Institutional Animal Care and Treatment Committee of Southwest Medical University (Luzhou, China).

### Main laboratory equipment and reagents

The following equipment and reagents were used in the study: linear accelerator (Elekta, Sweden), paraffin slicer (Leica instrument company, Germany), new BGZ series II type high precision oven (Shanghai boxun company, China), pipette (Eppende, Germany), inverted phase-contrast microscope (Olympus corporation, Japan), three-dimensional brachytherapy machine and oncentra brachytherapy treatment planning system (brachy TPS. Nucletron company, 4.3.0. Four hundred-ten version, The Netherlands), High-Glucose DMEM (HyClone, USA), fetal bovine serum (Gibco, USA), monoclonal antibodies against mouse CD4, CD8, Foxp3, CD86, Ki-67, and apoptosis detection reagent (Bio-World, USA), detection reagents for interleukin-10 (IL-10), IL-12, and interferon-γ (IFN-γ) [andyht (Beijing) company, China], micro-positron emission tomography/computer tomography (micro-PET/CT) system (Siemens, Germany), nanozoomer digital pathological section scanner (Hamamatsu photonics, Japan), and flow cytometer (BD, USA).

### Experimental grouping and specimen collection

The number of tumor-bearing mice in each group was determined by combining literature and statistics [20]. The tumor-bearing mice were numerically randomly divided into the control group [12 mice. Dt = 0 Gy (Gy)] and the experimental groups: (1) CFRT group [12 mice; radiation dose Dt = 20 Gy/10 fraction (F); 6 MV X-ray as radioactive source, source–skin distance = 100 cm] and (2) SBHFRT group (12 mice). The source applicator was implanted (1–2 needles) along the long axis of the tumor, simulated CT scan was performed, gross tumor volume (GTV) at brachy TPS was delineated, and the radiation plan was designed and implemented (brachytherapy dose Dt = 11.3 Gy/1 F, D_95%_ ≥ 10 Gy of GTV). The biologically equivalent dose (BED; tumor α / β = 10) was calculated according to the L-Q model in the CFRT and SBHFRT groups (BED = 24.00 Gy and 24.07 Gy, respectively).

**Fig. 1:**
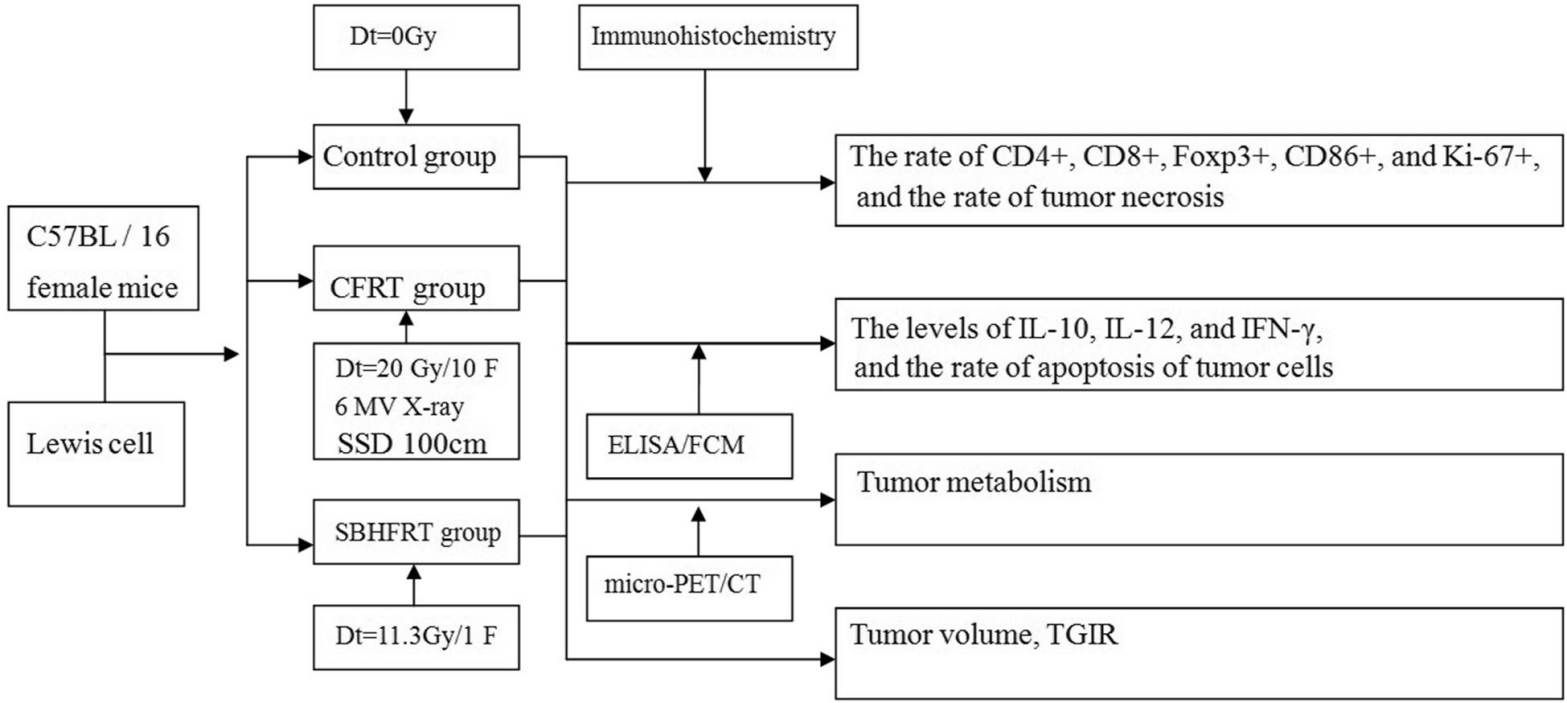
Research design and route. CFRT: conventional fraction radiotherapy; SBHFRT: single brachytherapy hypofractionated radiotherapy; ELISA: enzyme-linked immunosorbent assay; FCM: flow cytometry; IFN-γ: interferon-γ; IL-10: interleukin-10; TGIR: Tumor growth inhibition rate; micro-PET/CT: micro-positron emission tomography/computer tomography

On days 7 and 14 after radiation, six mice were euthanized by cervical dislocation in each group, and their tumors were completely stripped off, and averagely divided into two parts. One half was used for immunohistochemical analysis for detecting the positive expressions of CD4, CD8, Foxp3, CD86, and Ki-67 in cells, and the tumor necrosis rate. The other half was used for enzyme-linked immunosorbent assay (ELISA) and flow cytometry to detect levels of IL-10, IL-12, and IFN-γ, and the rate of apoptosis of tumor cells. The research design and route are shown in Fig. 1.

### Evaluation of CD4^+^, CD8^+^, Foxp3^+^, and CD86^+^ cells and Ki-67^+^ expression levels and calculation of tumor necrosis rate

The expression levels of CD4, CD8, Foxp3, CD86, and Ki-67 in tumor tissues were detected via immunohistochemical analysis following the SP method, and the results were independently evaluated by two experienced pathologists. Paraffin-embedded tumor tissues fixed using neutral formalin (10%) were cut into 3-μm thick slices. Paraffin was removed using xylene, and the latter was removed by alcohol immersion (75%). After rinsing the slices with water, they were put into slice boxes containing citratebuffersolution (pH 6.0) and processed in a microwave oven (500 W) for 5 min three times, and additional buffer was added after the second heating process. Subsequently, the slices were rinsed with Tris buffered saline (pH 7.6), and immersed in hydrogen peroxide (H_2_O_2_; 3%). After soaking, the slices were shaken and dried. Then, a circle was drawn along the tissue edge using a gel pen to keep the tumor tissue inside the circle. Following this, 5% fetal bovine serum was added into the circle, along with the consequent addition of CD4, CD8, Foxp3, CD86, and Ki-67 antibodies; the tissue slices were stored in a refrigerator at 4 °C overnight. The slices were cleaned with PBS solution on the next day, and secondary antibodies were added, followed by incubation at room temperature (37 °C) for 30 min. The slices were again cleaned with distilled water, followed by color rendering with the DAB liquid, and subsequently cleaned, dried, sealed, and observed under an optical microscope after reverse staining with hematoxylin.

The criteria for the evaluation of CD4^+^, CD8^+^, Foxp3^+^, and CD86^+^ cells were positive expressions of CD4, CD8, and CD86 on staining as shown by brown-yellow or brown cytoplasm or cell membrane. In contrast, the positive expressions of Foxp3 and Ki-67 were showed by brown-yellow or brown nucleus. Calculations of CD4^+^, CD8^+^, Foxp3^+^, and CD86^+^ cells were performed by selecting five fields for each section. The number of positive cells in each field was counted under a microscope (× 400), and the mean number of positive cells in the five fields was calculated as the number of positive cells in the section. The positive expression rate for Ki-67 was calculated by counting under a microscope (× 400). Five fields were selected in each section and the number of positive cells in each field was counted along with the total number of cells. The proportion of positive cells among the total cells in the five fields was calculated to determine the positive expression rate of Ki-67. The percent differences in the positive expression rates of CD4, CD8, Foxp3, CD86, and Ki-67 between groups were calculated using Formula 1.

Formula 1 $$ \mathrm{Ratio}\ \left(\%\right)=\left(\mathrm{rate}\ \mathrm{in}\ \mathrm{experimental}\ \mathrm{group}-\mathrm{rate}\ \mathrm{in}\ \mathrm{reference}\ \mathrm{group}\right)/\mathrm{rate}\ \mathrm{in}\ \mathrm{reference}\ \mathrm{group}\times 100\% $$

The stripped tumor tissue was fixed and dehydrated, and H&E staining sections were prepared and placed under Nanozoomer digital pathological section scanner to generate a full field of digital image, which was transmitted to the KFBIO, Slide Viewer image acquisition system. Subsequently, the tumor boundaries and necrotic areas were delineated, and the total tumor area (S_tumor_) and necrotic area (S_necrosis_) were calculated. Tumor necrosis rate was calculated using Formula 2. The percent difference in the tumor necrosis rate between groups was calculated using Formula 1.

Formula 2 $$ \mathrm{Tumor}\ \mathrm{necrosis}\ \mathrm{rate}={\mathrm{S}}_{\mathrm{necrosis}}/{\mathrm{S}}_{\mathrm{tumor}}\times 100\% $$

### Levels of IL-10, IL-12, and IFN-γ and tumor cell apoptosis

Levels of IL-10, IL-12, and IFN-γ in the tumor tissues were detected by ELISA, and the concentrations of IL-10, IL-12, and IFN-γ were calculated according to the standard curve. The differences in the ratios of IL-10, IL-12, and IFN-γ levels between different groups were calculated using Formula 1. Fresh tumor tissues added with PBS solution (pH 7.4), were ground into a homogenate, which was centrifuged at 2000 RPM for 20 min, and the supernatant was reserved. Reagents in the IL-10, IL-12, and IFN-γ testing kits were taken out. Subsequently, 10 empty standard wells were set on the enzyme-labeled coating plate, and 100 μL of the standard and 50 μL of the dilution sample were added to the wells 1 and 2, and mixed. Following this, 100 μL of the standard solution was pipetted from the two wells, and added into the adjacent wells 3 and 4; 50 μL of the dilution sample was also added to wells 3 and 4 and mixed well. Following this, 50 μL of the mixture from wells 3 and 4 was drained and discarded and 50 μL was added to wells 5 and 6 each and mixed well. This procedure was repeated for all wells. A blank well and sample well were set to be tested. Subsequently, 40 μL sample diluent and 10 μL supernatant were added to the sample well to be tested. After sealing the plate with a sealing dye, the plate was placed in an incubator at 37 °C for 30 min. Following this, the sealing plate film was removed, the liquid was discarded, and the washing liquid was added. After 30s, the sealing plate was discarded. The sample was incubated at 37 °C for 30 min. After washing, 50 μL of coloring agent A was added to each well, followed by 50 μL of coloring agent B. After shaking and mixing, the samples were incubated at 37 °C for 30 min. Subsequently, 50 μL of termination solution was added to the well of the sample to be tested. The wave length of the enzyme marker was set at 450 nm, and the absorbance (optical density; OD) value for each well was measured within 15 min. Using the MS Excel program, the linear regression equation of the standard curve was calculated based on the concentration and OD value of the standard substance, and the OD value of the sample was entered into the equation to calculate the sample concentration. The actual concentrations of IL-10, IL-12, and IFN-γ in the sample were thus obtained by multiplication with the dilution factor.

The apoptosis of tumor cells was analyzed via flow cytometry, and the apoptosis rate was calculated. The percent difference in the apoptosis rate between groups was calculated using Formula 1. A fresh tumor specimen was cut into pieces, washed twice with a sterile PBS (4 °C, pH 7.4) solution, and ground into a homogenate, which was then digested with trypsin for 20 min; the homogenate was then transferred into a 5-mL centrifuge tube and centrifuged at 2000 RPM for 5 min. Subsequently, the cell sediments at the bottom of the centrifuge tube were mixed into a uniform cell suspension, followed by centrifugation at 2000 RPM for 5 min, and the supernatant was discarded. Following this, 200 μL of Binding Buffer was added to the cell suspension, and the cell density was adjusted to 1 × 10^6^ /mL. This was followed by the addition of 5 μL of annexin V-FITC reagent into the flow cytometry tube, which had 195 μL of the cell suspension. After 5 min, 10 μL of PI reagent was added into the flow cytometry tube and mixed well, followed by incubation in dark at room temperature for 10 min. Subsequently, 300 μL sterile PBS solution was added to the flow cytometry tube, and mixed by horizontal shaking, and flow cytometry was performed.

### Tumor volume and tumor growth inhibition rate

The maximum (d_max_) and minimum diameters (d_min_) of transplanted tumors in each group were measured every 2 days from the 12th day after inoculation until the 14th day after radiation. Tumor growth inhibition rate (TGIR) was calculated on the 14th day after radiation. Tumor volume and TGIR were calculated using Formulas 3 and 4. The percent differences of tumor volume and TGIR rate between groups were calculated using Formula 1.

Formula 3 $$ \mathrm{Tumor}\ \mathrm{volume}\left({\mathrm{V}}_{\mathrm{tumor}}\right)=\left({\mathrm{d}}_{\mathrm{max}}\times {{\mathrm{d}}_{\mathrm{min}}}^2\right)/2 $$

Formula 4 $$ \mathrm{TGIR}=\left(\mathrm{control}\ \mathrm{group}\ {\mathrm{V}}_{\mathrm{tumor}}-\mathrm{experimental}\ \mathrm{group}\ {\mathrm{V}}_{\mathrm{tumor}}\right)/\mathrm{control}\ \mathrm{group}\ {\mathrm{V}}_{\mathrm{tumor}}\times 100\% $$

### Micro-PET/CT

On days 7 and 14 after radiation, six mice from each group underwent 18 F-FDG micro-PET/CT (parameters 80 Kv, 500 uA; Space 1.5 mm). The images of micro-PET/CT were analyzed by two experienced physicians in the Nuclear Medicine Department, and the maximum standardized uptake value (SUV_max_) of tumor tissues was calculated. The percent difference of SUV_max_ rate between groups was calculated using Formula 1.

### Statistical analysis

SPSS 17.0 software was used for statistical analysis, and the measurement data are expressed as mean ± S. One-way analysis of variance (ANOVA) and least significant difference (LSD)-t test were used for comparisons between groups. TGIR was compared by t test, and *p* < 0.05 indicated that the difference was statistically significant.

## Results

### Tumor volume, TGIR, and SUV_max_

**Fig. 2:**
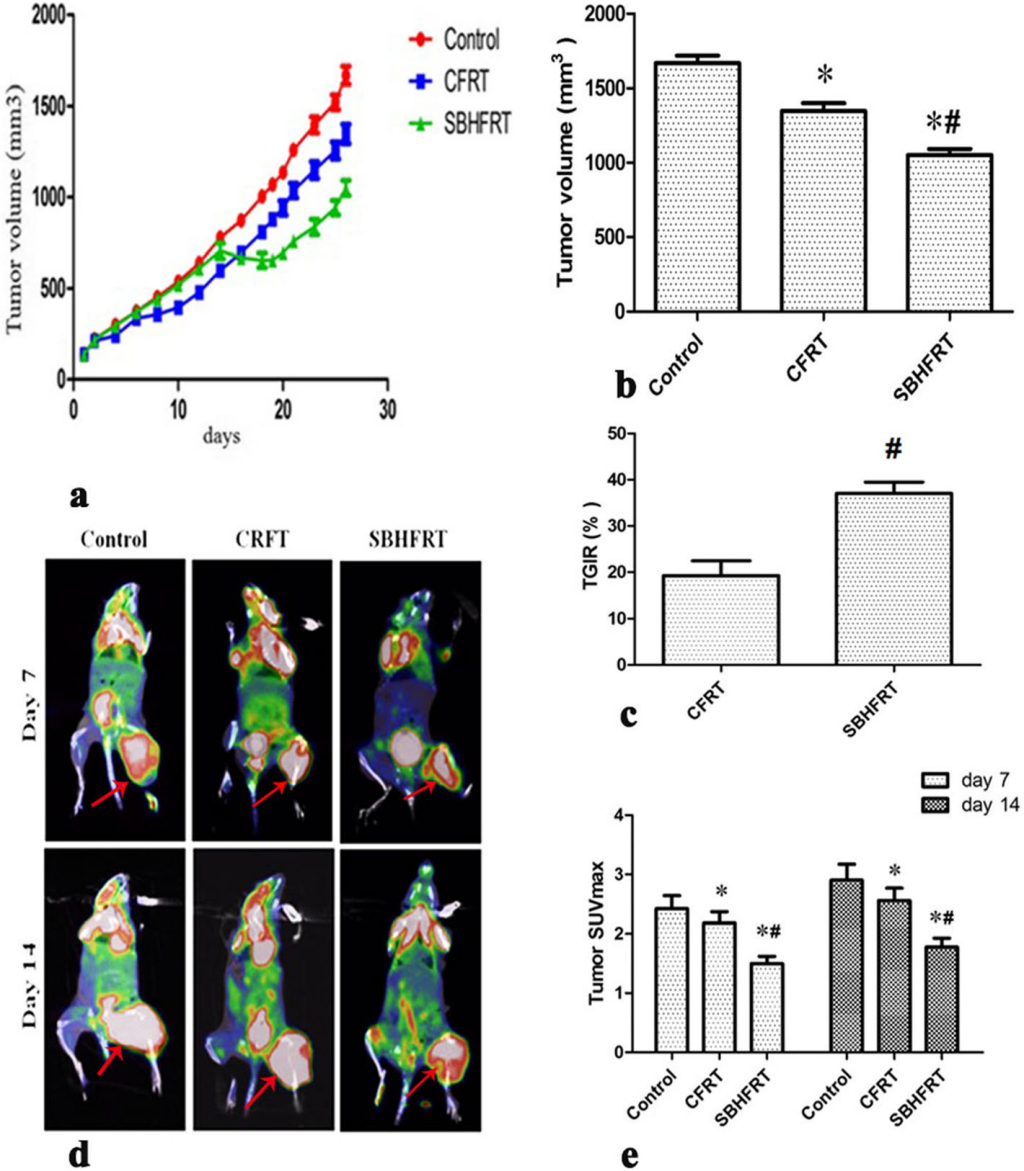
Changes in tumor status and tumor metabolic activity as evaluated by Micro-PET/CT between groups and at different time points. On day 18, the tumor growth in SBHFR group was significantly lower than that in CFRT and control groups (a). Tumor volume (b. F = 242.94, *p* < 0.05) and tumor growth inhibition rate (c. t = 10.70, *p* < 0.05) in SBHFR group were significantly lower than those in CFRT and control groups. The tumor metabolic range in SBHFR group was the smallest on day 7 (d. Red arrow), and the tumor metabolic activity in SBHFR group was significantly lower than that in CFRT and control groups on both days 7 and 14 (e. F = 42.60 on day 7 and 43.03 on day 14; *p* < 0.05). The difference was the most obvious on day 7. * Compared with the control group. # Compared with the CFRT group

The tumor volume in the SBHFRT group reduced by 22.0 and 37.0% compared with that in the CFRT and control groups, respectively (*p* < 0.05), whereas the volume in the CFRT group reduced by 19.2% compared with that in the control group (*p* < 0.05). TGIR in the SBHFRT group increased by 92.2% compared with that in the CFRT group (t = 10.70, *p* < 0.05). SUV_max_ in the SBHFRT group was lower than that in the CFRT and control groups (*p* < 0.05) on days 7 and 14 after radiation, whereas SUV_max_ in the CFRT group was only lower by 8.3 and 10.3% compared with that in the control group, respectively (*p* < 0.05). Furthermore, the difference was most obvious on day 7 after radiation(t = 3.57, 3.29; *p* < 0.05). Figure 2; Table 1.

**Table 1:** Comparisons of tumor volume, SUV_max_, TGIR, tumor necrosis rate, apoptosis rate, and Ki-67^+^ expression rate among groups and at different time points (mean ± S)

Groups	*n*	Tumor necrosis rate		Apoptosis rate		Rate of Ki-67^+^		V_tumor_ (mm^3^)	SUV_max_ value		TGIR^§^ (%)
		Day 7 (ratio, %)	Day 14 (ratio, %)	Day 7 (ratio, %)	Day 14 (ratio, %)	Day 7 (ratio, %)	Day 14 (ratio, %)		Day 7 (ratio, %)	Day 14 (ratio, %)	
Control	6	18.3 ± 3.6	16.0 ± 3.8	3.4 ± 0.3	2.8 ± 0.4	79.0 ± 5.6	89.5 ± 4.5	4169.8 ± 123.3	2.4 ± 0.2	2.9 ± 0.3	
CFRT^*^	6	23.1 ± 4.6 (+^↑^26.2^△^)	18.9 ± 4.0 (+ 18.1^△^)	3.9 ± 0.4 (+ 14.7^△^)	3.2 ± 0.5 (+ 14.3^△^)	72.5 ± 9.1 (−^↓^8.2^△^)	81.0 ± 5.8^#^ (− 9.5^△^)	3368.7 ± 134.9^#^	2.2 ± 0.2^#^ (− 8.3^△^)	2.6 ± 0.2^#^ (− 10.3^△^)	19.2 ± 3.2
SBHFRT^Ұ^	6	51.8 ± 7.1^#※^ (+ 183.1^△^ /+ 124.2^▼^)	47.4 ± 6.8^#※^ (+ 196.3^△^ /+ 150.8^▼^)	7.3 ± 0.7^#※^ (+ 114.7^△^ /+ 87.2^▼^)	5.7 ± 0.7^#※^ (+ 103.6^△^ /+ 78.1^▼^)	41.8 ± 4.6^#※^ (− 47.1^△^ /− 42.3^▼^)	44.8 ± 7.2^#※^ (− 49.9^△^ /− 44.7^▼^)	2627.3 ± 103.4^#※^	1.5 ± 0.1^#※^ (− 37.5^△^ /− 31.8^▼^)	1.8 ± 0.2^#※^ (− 37.9^△^ /− 30.8^▼^)	37.0 ± 2.5^※^
F		70.22	71.28	114.06	52.31	52.12	95.83	242.94	42.60	43.03	10.70(t)
P		0.00	0.00	0.00	0.00	0.00	0.00	0.00	0.00	0.00	0.00

^*^Conventional fractionation radiotherapy

^Ұ^Single brachytherapy hypofractionated radiotherapy

^§^Tumor growth inhibition rate

^#^Compared with the control group, *p* < 0.05

^※^Compared with theCFRT group, *P* < 0.05

^△^Ratiocompared with the control group

^▼^Ratiocompared with the CFRT group

^↑^Increasing

^↓^Decreasing

### Tumor cell necrosis and apoptosis rates and Ki-67^+^ expression rate

**Fig. 3:**
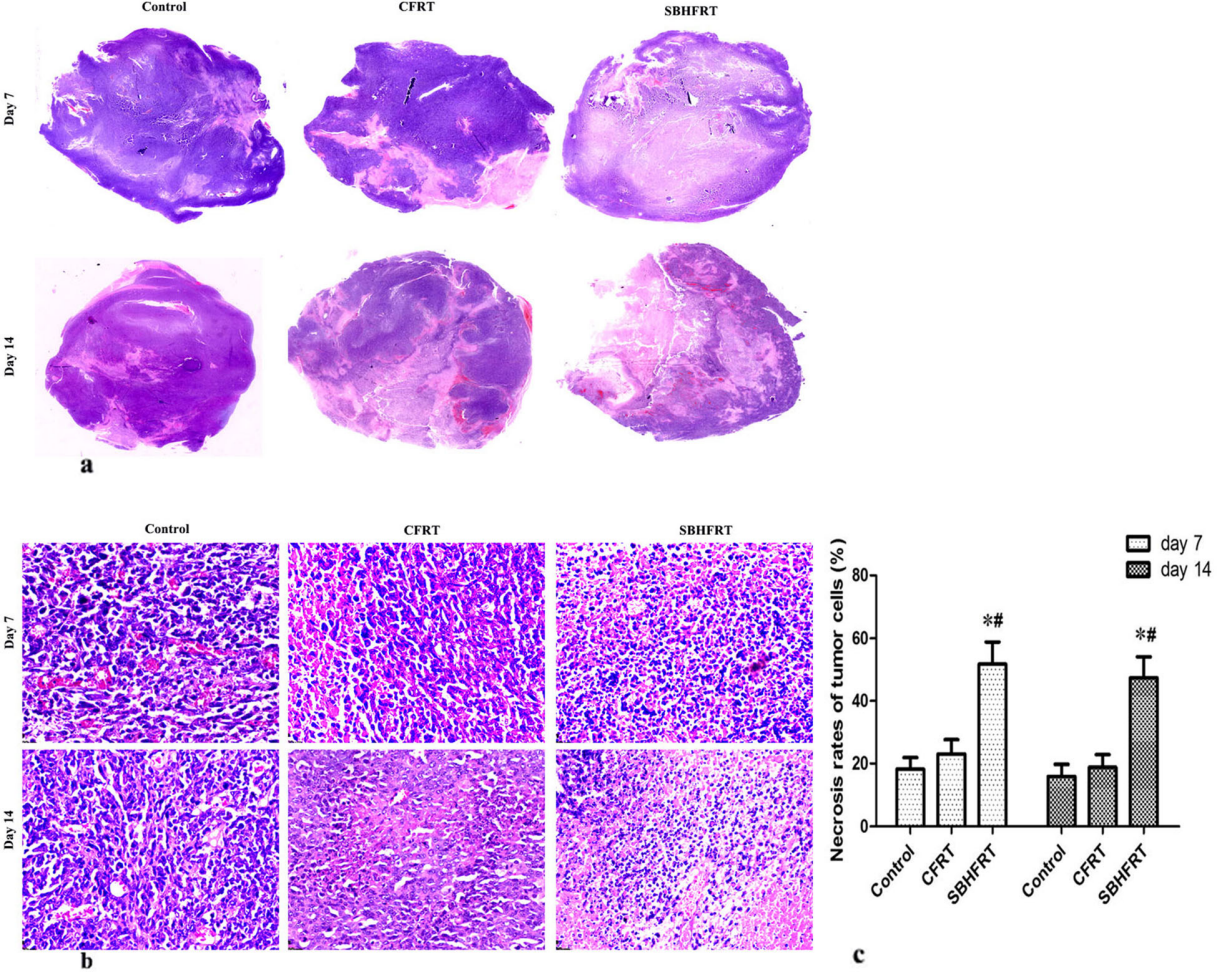
Tumor tissue necrosis among groups and at different time points. Tumor scan was collected using KFBIO, Slide Viewer image capture system **a**. H&E staining **b**. The tumor necrosis rate in SBHFRT group was higher than that in CFRT and control groups on days 7 (F = 70.22, *p* < 0.05) and 14 (F = 71.28, *p* < 0.05). The difference was the most obvious on day 7 **c**. * Compared with the control group. # Compared with the CFRT group

**Fig. 4:**
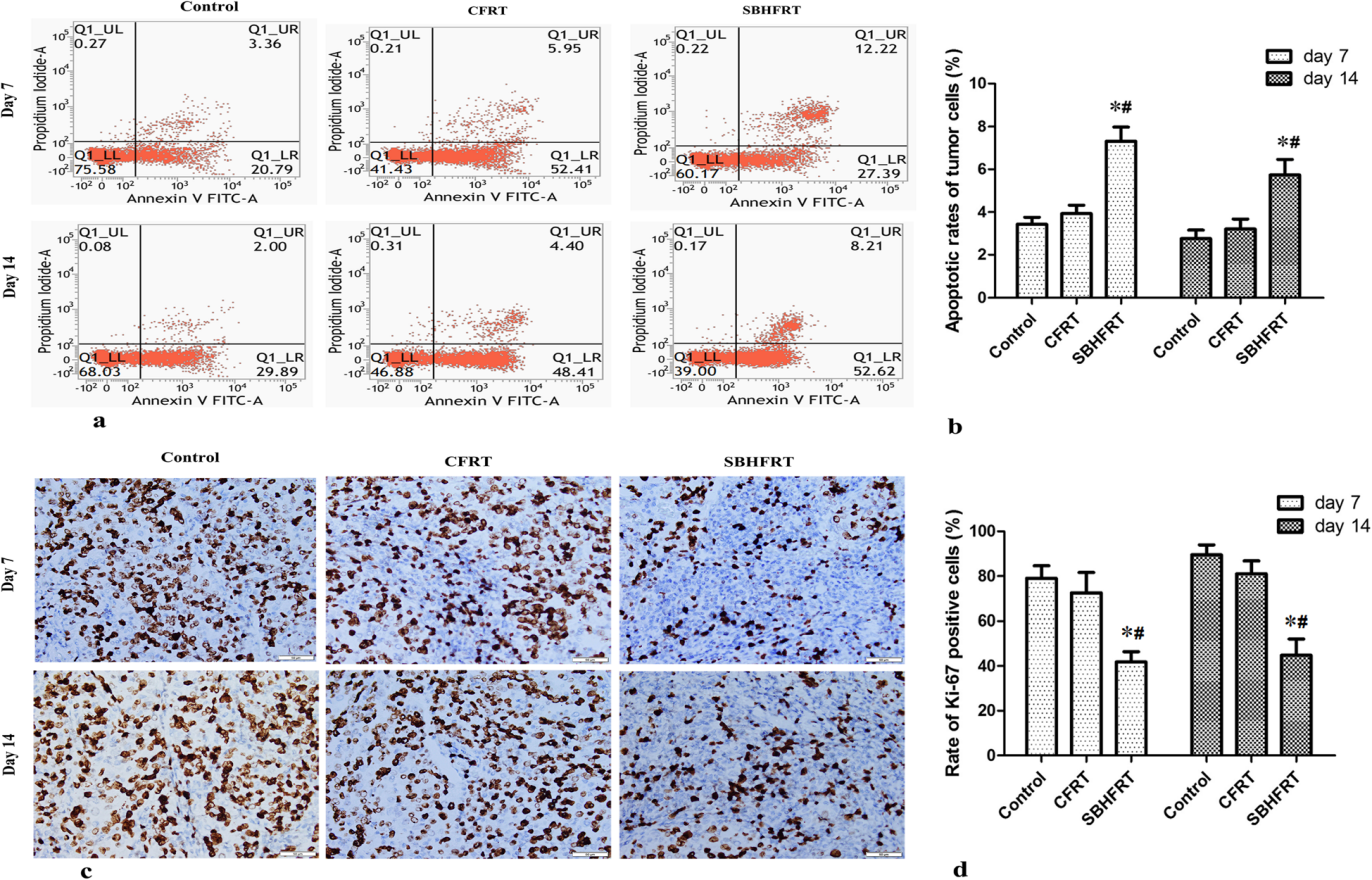
Tumor cell apoptosis and positive expression of Ki-67 (shown by brown-yellow or brown particles; × 400) among groups and at different time points. Tumor cell apoptosis rate in the SBHFRT group was significantly higher than that in the CFRT and control groups **a**, **b**. F = 114.06 on day 7 and 52.31 on day 14; *p* < 0.05), and the positive expression rate of Ki-67 in the SBHFRT group was significantly lower than that in the CFRT and control groups **c**, **d** on days 7 and 14 (F = 52.12 on day 7 and 95.83 on day 14; *p* < 0.05). The difference was the most obvious on day 7.* Compared with the control group. # Compared with the CFRT group

Tumor cell necrosis and apoptosis rates in the SBHFRT group were higher than those in the CFRT and control groups on days 7 and 14 after radiation (*p* < 0.05), whereas there were no significant differences in the rates in the CFRT group compared with those in the control group (*p* > 0.05). The most obvious difference in apoptosis rates was observed on day 7 after radiation (t = 3.91, 2.93; *p* < 0.05). Figures 3, 4; Table 1.

Ki-67^+^ expression rates in the SBHFRT group were lower than those in the CFRT and control groups on days 7 and 14 after radiation (*p* < 0.05). However, there were no differences between the expression rates in the CFRT and control groups on day 7 and those on day 14 (*p* > 0.05). Figure 4; Table 1.

### Expression rates of CD86^+^, CD4^+^, CD8^+^, and Foxp3^+^ cells

**Fig. 5:**
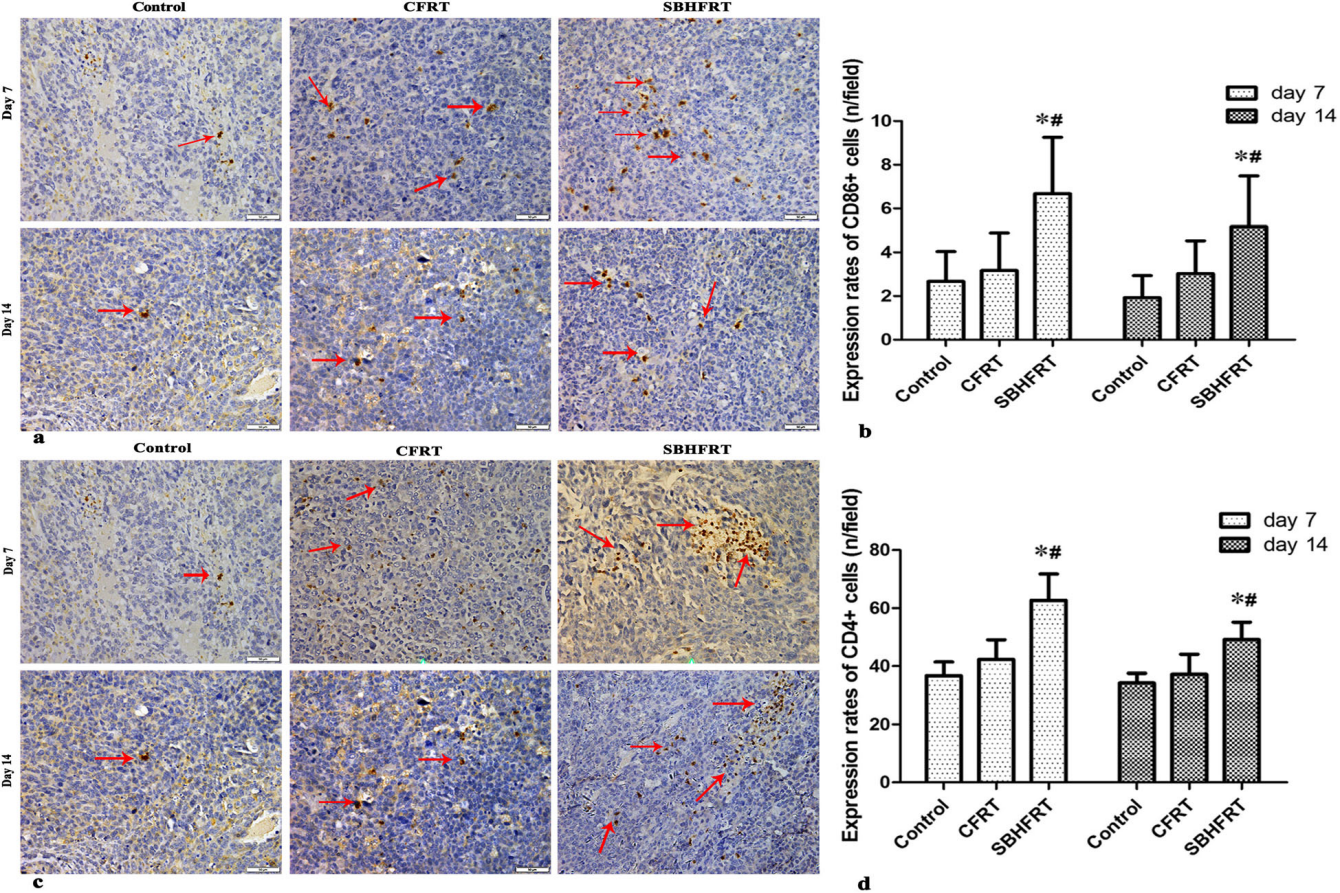
Positive expressions of CD86 and CD4 among groups and at different time points (shown by brown-yellow or brown particles; × 400; red arrow). The positive expression rates of CD86 **a**, **b**. F = 7.44 on day 7 and 5.63 on day 14; *p* < 0.05) and CD4 (**c**, **d**. F = 22.24 on day 7 and 11.89 on day 14; *p* < 0.05) in the SBHFR group were significantly higher than those in the CFRT and control groups on days 7 and 14. The difference was the most obvious on day 7. * Compared with the control group. # Compared with the RT group

**Fig. 6:**
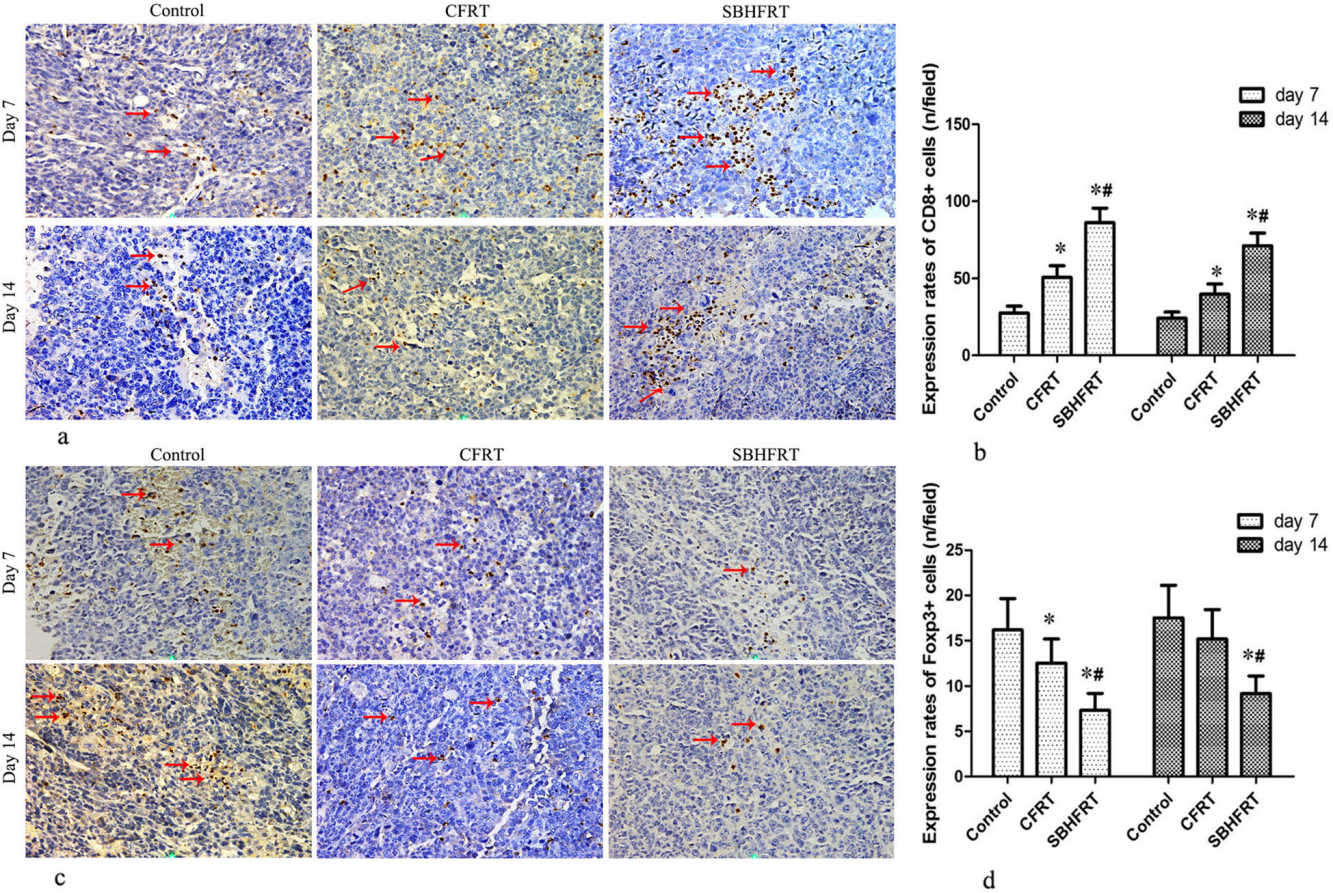
Positive expressions of CD8 and Foxp3 among groups and at different time points (shown by brown-yellow or brown particles; × 400; red arrow). The positive expression rates of CD8 **a**, **b**. F = 92.24 on day 7 and 82.05 on day 14, *p* < 0.05) in the SBHFR group was significantly higher than that in the CFRT and control groups, whereas the positive expression rate of Foxp3 in the SBHFR group was significantly lower than that in the CFRT and control groups on days 7 and 14 **c**, **d**. F = 15.59 on day 7 and 12.13 on day 14; *p* < 0.05). The difference was the most obvious on day 7. * Compared with the control group. # Compared with the RT group

The rates of CD86^+^, CD4^+^ and CD8^+^ cells in the SBHFRT group were higher than those in the CFRT and control groups on days 7 and 14 after radiation (*p* < 0.05). The most obvious differences were observed on day 7 after radiation (*p* < 0.05). There were higher differences in the rates of CD4^+^ and CD8^+^ cells on day 7 than on day 14 in the SBHFRT group (t = 3.04, 2.85; *p* < 0.05). Figures 5, 6;Table 2.

**Table 2:** Comparisons of the expression rates of CD86^+^, CD4^+^, CD8^+^, and Foxp3^+^ cells in tumor tissues among groups and at different time points (rate/field; mean ± S)

Groups	n	Rate of CD86^+^ cells		Rate of CD4^+^ cells		Rate of CD8^+^ cells		Rate of Foxp3^+^ cells	
		Day 7 (ratio,%)	Day 14 (ratio,%)	Day 7 (ratio,%)	Day 14 (ratio,%)	Day 7 (ratio,%)	Day 14 (ratio,%)	Day 7 (ratio,%)	Day 14 (ratio,%)
Control	6	2.7 ± 1.4	1.9 ± 1.0	36.8 ± 4.6	34.3 ± 3.3	27.5 ± 4.5	24.2 ± 4.0	16.2 ± 3.5	17.5 ± 3.6
CFRT^*^	6	3.2 ± 1.7 (+^↑^18.5^△^)	3.0 ± 1.5 (+ 57.9^△^)	42.3 ± 6.8 (+ 14.9^△^)	37.3 ± 6.8 (+ 8.7^△^)	50.7 ± 7.6^#^ (+ 84.4^△^)	39.8 ± 6.6^#^ (+ 64.5^△^)	12.5 ± 2.7^#^ (−^↓^22.8^△^)	15.2 ± 3.3 (− 13.1^△^)
SBHFRT^Ұ^	6	6.7 ± 2.6^#※^ (+ 148.1^△^/ + 109.4^▼^)	5.2 ± 2.3^#※^ (+ 173.7^△^/ + 73.3^▼^)	62.7 ± 9.1^#※^ (+ 70.4^△^/ + 48.2^▼^)	49.2 ± 6.0^#※^ (+ 43.4^△^/ + 31.9^▼^)	86.0 ± 9.6^#※^ (+ 212.7^△^/ + 69.6^▼^)	71.3 ± 8.2^#※^ (+ 194.6^△^/ + 79.1^▼^)	7.3 ± 1.9^#※^ (− 54.9^△^/ -41.6^▼^)	9.2 ± 1.9^#※^ (− 47.4^△^/ -39.5^▼^)
*F*		7.44	5.63	22.24	11.89	92.24	82.05	15.6	12.13
*P*		0.01	0.02	0.00	0.00	0.00	0.00	0.00	0.00

^*^Conventional fractionation radiotherapy

^Ұ^Single brachytherapy hypofractionated radiotherapy

^#^Compared with the control group, *p* < 0.05

^※^Compared with theCFRT group, *p* < 0.05

^△^Ratiocompared with the control group

^▼^Ratiocompared with the CFRT group

^↑^Increasing

^↓^Decreasing

The rates of Foxp3^+^ cells in the SBHFRT group were lower than those in the CFRT and control groups on days 7 and 14 after radiation (*p* < 0.05). Further, the rate of Foxp3^+^ cells in the CFRT group was lower than that in the control group (p < 0.05). The most obvious difference was on day 7. There were no differences between day 7 and day 14 in the SBHFRT and CFRT groups (t = 1.68, 1.56; *p* > 0.05). Figure 6, Table 2.

### Levels of IL-10, IL-12, and IFN-γ in tumor tissues

**Fig. 7:**
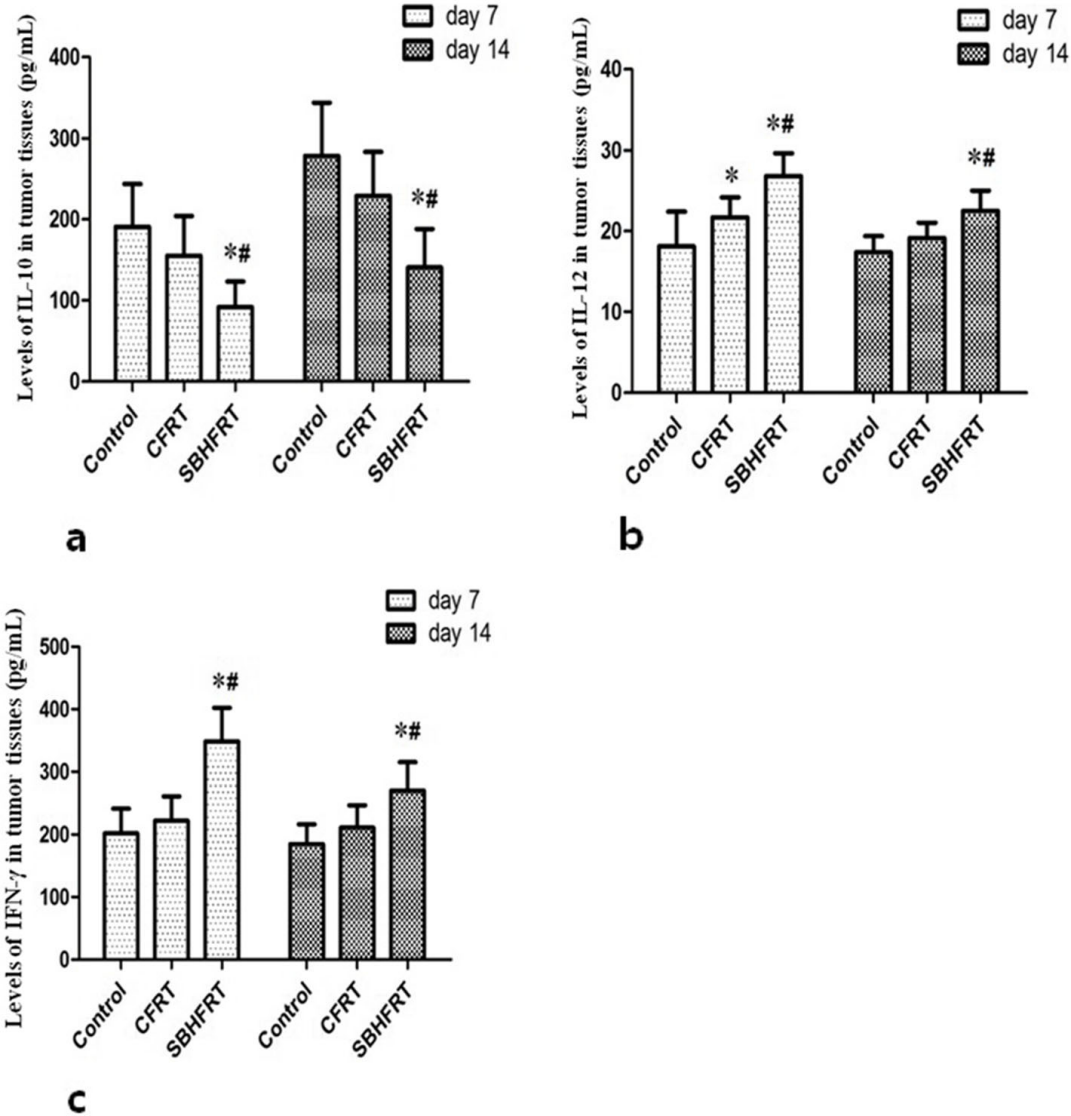
Levels of IL-10, IL-12, and IFN-γ in tumor tissues among groups and at different time points. The level of IL-10 in SBHFR group was significantly lower than that in the CFRT and control groups (**a**. F = 7.38 on day 7 and 9.32 on day 14; *p* < 0.05), whereas the levels of IL-12 (**b**. F = 19.68 on day 7 and 8.70 on day 14; *p* < 0.05) and IFN-γ (**c**. F = 18.94 on day 7 and 8.11 on day 14; *p* < 0.05) in SBHFR group were significantly higher than those in the of CFRT and control groups on days 7 and 14. The difference was the most obvious on day 7. * Compared with the control group. # Compared with the RT group

IL-10 levels in the SBHFRT group were lower than the levels in the CFRT and control groups on days 7 and 14 after radiation (*p* < 0.05). The most obvious difference was on day 7. The IL-10 levels were not different between the CFRT and control groups (p > 0.05). However, there were differences observed between days 7 and 14 in the SBHFRT and CFRT groups (t = 2.55, 2.49; *p* < 0.05). Figure 7, Table 3.

**Table 3:** Comparisons of IL-10, IL-12, and IFN-γ levels in tumor tissues among groups and at different time points (pg/mL, mean ± S)

Groups	n	IL-10 levels		IL-12 levels		IFN-γ levels	
		Day 7 (ratio,%)	Day 14 (ratio,%)	Day 7 (ratio,%)	Day 14 (ratio,%)	Day 7 (ratio,%)	Day 14 (ratio,%)
Control	6	190.9 ± 53.0	257.4 ± 65.4	18.1 ± 4.3	17.4 ± 2.0	201.9 ± 39.7	184.9 ± 31.6
CFRT^*^	6	155.3 ± 48.8 (−^↓^18.6^△^)	219.9 ± 54.1 (− 14.6^△^)	21.7 ± 2.5# (+^↑^19.9^△^)	19.1 ± 1.9 (+ 9.8^△^)	222.2 ± 38.8 (+ 10.1^△^)	210.8 ± 36.1 (+ 14.0^△^)
SBHFRT^Ұ^	6	91.6 ± 31.5^#※^ (− 52.0^△^ /− 41.0^▼^)	135.8 ± 47.6^#※^ (− 47.2^△^ /− 38.2^▼^)	26.8 ± 2.8^#※^ (+ 48.1^△^ /+ 23.5^▼^)	22.5 ± 2.5^#※^ (+ 29.3^△^ /+ 17.8^▼^)	348.4 ± 53.9^#※^ (+ 72.6^△^ /+ 56.8^▼^)	270.7 ± 44.6^#※^ (+ 46.4^△^ /+ 28.4^▼^)
*F*		7.38	7.38	19.68	8.70	18.94	8.11
*P*		0.01	0.01	0.00	0.00	0.00	0.00

^*^Conventional fractionation radiotherapy

^Ұ^Single brachytherapy hypofractionated radiotherapy

^#^Compared with the control group, *p* < 0.05

^※^Compared with theCFRT group, *p* < 0.05

^△^Ratiocompared with the control group

^▼^Ratiocompared with the CFRT group

^↑^Increasing

^↓^Decreasing

IL-12 and IFN-γ levels in the SBHFRT group were higher than those in the CFRT and control groups on days 7 and 14 after radiation (*p* < 0.05). The most obvious differences were observed on day 7. IL-12 and IFN-γ levels in the CFRT group were considerably higher than those in the control group (*p* < 0.05). Differences in levels between days 7 and 14 were only observed in the SBHFRT group (t = 2.79, 2.72; *p* < 0.05). Figure 7, Table 3.

## Discussion

Radiation induces tumor cell DNA damage and death, and leads to in-situ vaccination, promoting DCs, antitumor lymphocytes, and cytokines to accumulate in tumor tissues [21]. The quantity of in-situ vaccine produced by radiation is closely associated with tumor cell death. The result of our study indicated that under the same BED, the tumor showed a larger decrease in volume after SBHFRT than after CFRT, and TGIR after SBHFRT was nearly twice that after CFRT. On days 7 and 14 after radiation, SUV_max_ and Ki-67^+^ expression rates showed a larger decrease after SBHFRT than after CFRT. Further, tumor necrosis and apoptosis rates were considerably higher after SBHFRT than after CFRT on days 7 and 14. This indicated that SBHFRT was more effective than CFRT in terms of the physical role of radiation, and could result in a higher rate of tumor cell death, and lead to a high level of in-situ vaccination.

The relationship between radiation and immunity is complex. Immunologic effects induced by radiation are not only sensitive to variations in dose and fractionation but are also sensitive to time [21]**.** The results of a comparative study with a 48 Gy/8 F or 48 Gy/6 F regimen for treatment by SBRT in NSCLC patients indicated that the levels of CD3^+^ T lymphocytes were slightly higher after SBRT, and they significantly increased at three weeks. The ratio of CD8^+^ T cells / CD3^+^ T cells increased significantly three weeks after SBRT [18]. In our study, we selected days 7 and 14 as the observation time windows, and it was possible to observe the high expression of tumor invasion lymphocytes and cytokines and the relationship between hypofractionated radiotherapy and tumor immune microenvironment.

Radiation can enhance the efficacy of immune checkpoint blockers and promote immune responses, but the dose and fraction mode of radiation that induces this immune effect is unclear, and the recruitment of DCs is closely related to radiation dose and fraction mode [22]. The co-stimulatory factor CD86 is a surface marker of mature DCs [23]. Colon cancer studies have shown that 48 h after 10 Gy/F radiation, mature CD86^+^ DCs in tumor tissues significantly increased [24]. In our study, on days 7 and 14 after radiation, the number of CD86^+^ cells showed a higher increase in the SBHFRT group than that in the CFRT group. On comparing the results on days 7 and 14, the number of CD86^+^ cells in the SBHFRT group was higher on day 7 than on day 14; the number of CD86^+^ cells was higher on day 7 in the CFRT group too, but only by 6.7%. The reason may be the increased release of radiation-associated antigenic proteins induced by SBHFRT. SBHFRT can lead to a higher accumulation of mature DCs compared with CFRT, especially on day 7, with radiation-induced dead tumor cells acting as antigens to play a more effective role in antigen presentation.

Lymphocytes and cytokines in the tumor immune microenvironment play an important role in anti-tumor immunity [16, 25, 26]. T cells are the main cell type in the tumor microenvironment. Without intervention, CD4^+^ T cells and T reg cells are the main cell types, and the level of CD8^+^ T cells is usually very low [27]. An increase in the number of anti-tumor lymphocytes and cytokines in the tumor microenvironment can enhance the anti-tumor immune response [16, 17]. Studies have shown that high expression of CD4^+^ and CD8^+^ T lymphocytes in NSCLC led to the increase in the median survival and 3-year survival rates rather than low expression and no expression, whereas low expression of Foxp3^+^ T lymphocytes led to higher median survival and 3-year survival rates rather than high expression [28]. In our study, on days 7 and 14 after radiation, the expressions of CD4^+^ and CD8^+^ cells in the SBHFRT group were higher than those in the CFRT group; further, the expression of Foxp3^+^ T lymphocytes was lower in the SBHFRT group than in the CFRT group. In the SBHFRT group, the rate of increase of CD4^+^ and CD8^+^ cells on day 7 was higher than that on day 14, and the rate of decrease of Foxp3^+^ cells was higher than that on day 14. This may be because SBHFRT rather than CFRT can induce higher expression levels of T helper lymphocytes and cytotoxic T lymphocytes, especially 7 days after radiation; promote the accumulation of the anti-tumor cytokine IFN-γ [29]; regulate T lymphocyte decrease, which is beneficial to reduce tumor cell immunosuppression; and recruit more DCs and cytotoxic T lymphocytes, enhancing tumor cell antigen recognition, and in turn having a stronger antitumor effect [27, 29].

**Fig. 8:**
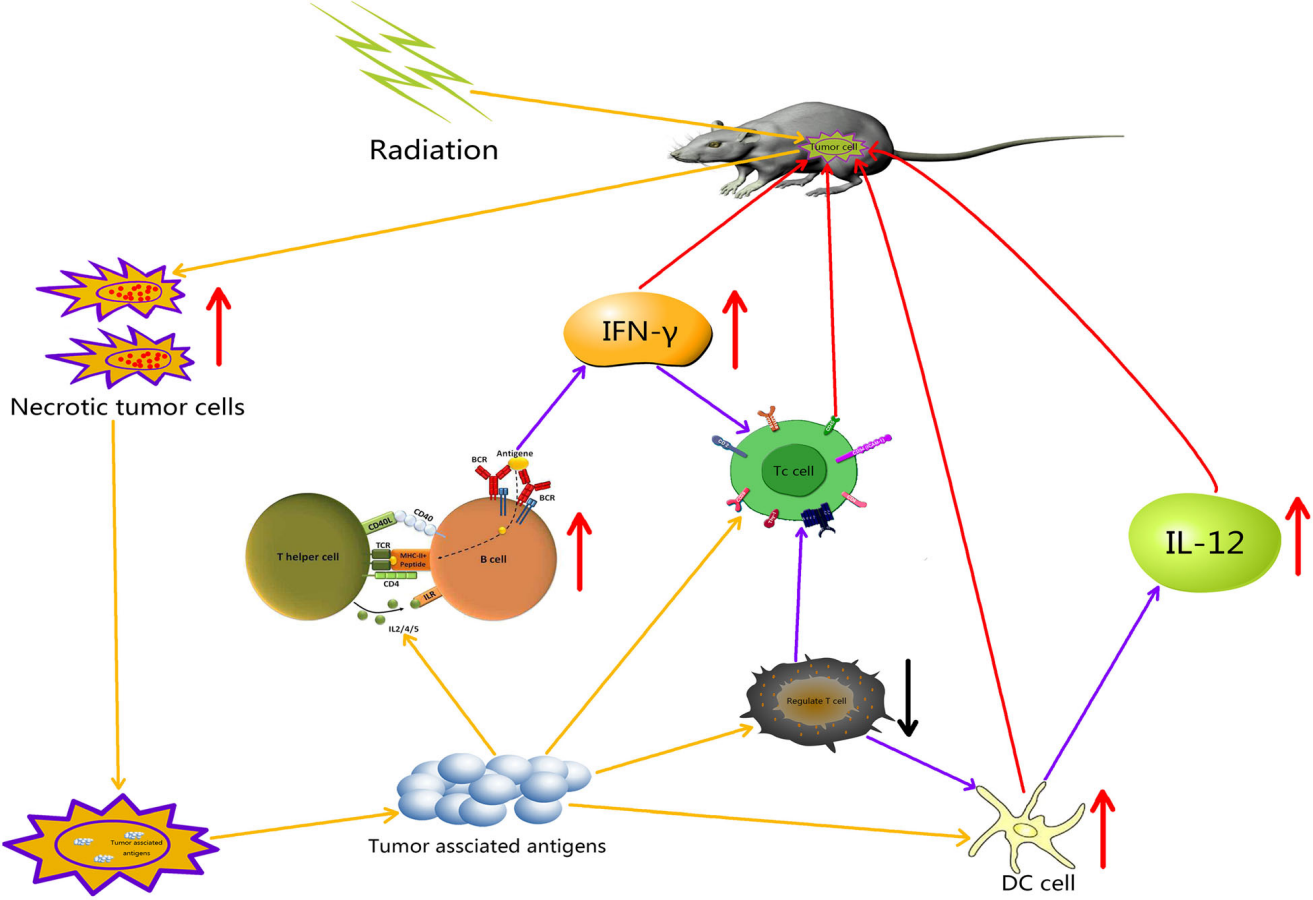
The possible mechanisms of SBHFRT effects on lymphocytes and cytokines in tumor microenvironment. IFN-γ: interferon-γ; DCs: dendritic cells. IL-12: interleukin-12. Positive effect (orange arrow). Acceleration effect (purple arrow). Up-regulation (red arrow). Down-regulation (black arrow). Feedback antitumor effect (red arrow)

Cytokines also play an important role in the anti-tumor immune response. IL-12 can induce the production of IFN-γ, stimulate the proliferation and activation of CD8^+^ T lymphocytes, and play a role in promoting the anti-tumor immune response [30, 31]. As an immunosuppressive factor, IL-10 can inhibit not only the apoptosis of tumor cells but also the role of IFN-γ and the anti-tumor immune response [30, 32]. In our study, on days 7 and 14 after radiation, IL-12 and IFN-γ levels in the SBHFRT group were obviously higher than those in the CFRT group, and IL-10 level was obviously lower than that in the CFRT group. On day 7, the rate of increase of IL-12 and IFN-γ levels, and the rate of decrease of IL-10 level in the SBHFRT group were significantly higher than those on day 14. This may be because SBHFRT resulted in a high rate of tumor cell necrosis in a short term; released a large number of tumor-associated antigens; led to a higher accumulation of DCs to secrete higher levels of IL-12; activated cytotoxic T lymphocytes and CD4^+^ T cells; produced more IFN-γ, promoting cytotoxic T cell proliferation and activation; and played a role in the positive feedback to adjust the secretion of IL-12 by DCs, while reducing the secretion of IL-10, which is beneficial for the generation of IL-12 and IFN-γ [31, 33, 34]. The possible mechanisms are shown in Fig. 8.

## Conclusion

SBHFRT could lead to a higher accumulation of DCs, anti-tumor lymphocytes and cytokines, and reduce the aggregation of immunosuppressive lymphocytes and cytokines in the tumor tissue compared with CFRT; this difference was the most obvious on day 7 after radiation. However, the clinical significance of this finding remains to be further verified. One of the limitations of the study is that we did not design the evaluation in a variety of cell lines. More detailed and in-depth studies, including Western blotting studies to detect the expression levels of proteins and studies in a variety of animal models and cell lines, should be performed in the future.

## Data Availability

The datasets used and/or analyzed during the current study are available from the corresponding author on reasonable request.

## Abbreviations

NSCLC: Non-small cell lung cancer
CFRT: Conventional fraction radiotherapy
SBRT: Stereotactic body radiotherapy
IFN-γ: Interferon-γ
DCs: Dendritic cells
SBHFRT: Single brachytherapy hypofractionated radiotherapy
IL-10: Interleukin-10
micro-PET/CT: Micro-positron emission tomography/computer tomography
F: Fraction
GTV: Gross tumor volume
BED: Biologically equivalent dose
ELISA: Enzyme-linked immunosorbent assay
d: Diameter
V_tumor_: Tumor volume
TGIR: Tumor growth inhibition rate
SUV: Standardized uptake value
